# Mercury Dynamics in the Sea of Azov: Insights from a Mass Balance Model

**DOI:** 10.3390/toxics12060417

**Published:** 2024-06-07

**Authors:** Christoph Gade, Rebecca von Hellfeld, Lenka Mbadugha, Graeme Paton

**Affiliations:** 1 National Decommissioning Centre, University of Aberdeen, Aberdeen AB41 6AA, UK; 2 School of Biological Sciences, University of Aberdeen, Cruickshank Building, St. Machar Drive, Aberdeen AB24 3UU, UK

**Keywords:** HERMES, environmental fate, speciation modelling, environmental monitoring

## Abstract

The Sea of Azov, an inland shelf sea bounding Ukraine and Russia, experiences the effects of ongoing and legacy pollution. One of the main contaminants of concern is the heavy metal mercury (Hg), which is emitted from the regional coal industry, former Hg refineries, and the historic use of mercury-containing pesticides. The aquatic biome acts both as a major sink and source in this cycle, thus meriting an examination of its environmental fate. This study collated existing Hg data for the SoA and the adjacent region to estimate current Hg influxes and cycling in the ecosystem. The mercury-specific model “Hg Environmental Ratios Multimedia Ecosystem Sources” (HERMES), originally developed for Canadian freshwater lakes, was used to estimate anthropogenic emissions to the sea and regional atmospheric Hg concentrations. The computed water and sediment concentrations (6.8 ng/L and 55.7 ng/g dw, respectively) approximate the reported literature values. The ongoing military conflict will increase environmental pollution in the region, thus further intensifying the existing (legacy) anthropogenic pressures. The results of this study provide a first insight into the environmental Hg cycle of the Sea of Azov ecosystem and underline the need for further emission control and remediation efforts to safeguard environmental quality.

## 1. Introduction

Mercury (Hg) is a highly toxic, environmentally persistent, and bioaccumulating element that poses a significant hazard to the marine biome [1]. Although it is naturally abundant and globally omnipresent, local concentrations tend to be highly spatiotemporally variable and depend on geographic location and surrounding industrial activity [2]. The Sea of Azov (SoA) is bounded by Ukraine and Russia and represents an important watershed due to its high abundance of fish and connection to the Black Sea [3]. However, due to the intense industry in the adjacent metropolises, the SoA has been heavily impacted by anthropogenic pollution, including Hg [4,5,6].

In May 2023, Ukraine accessed the Minamata Convention, which aims to control the supply and trade of Hg and reduce its use, emissions, and release [7]. Due to a historic lack of investment in environmental protection measures and the high density of heavy industries within the region, the levels of air and water pollution in the south and east of the country are amongst the highest in Europe [8]. As a signatory since 2014, Russia has also committed to implementing the agreements of the Minamata Convention and has reportedly already made significant progress in reducing Hg loadings in surface watersheds [9,10]. However, both countries continue to be affected by legacy pollution associated with coal-fired power stations and the historical use of organomercury-containing pesticides [11,12].

Mercury is a complex contaminant due to its intrinsic elemental properties, which cause it to readily speciate and alter its chemical behaviour [13,14]. Speciation is defined as ‘the distribution of the element among various chemical forms, which together make up the total concentration of the element in the system’ [15]. Elemental mercury (Hg0) possesses a low melting temperature and high vapour pressure, which facilitate volatilisation [16]. Volatilised Hg can remain in the atmosphere for months before being redeposited through rainfall, becoming a significant pathway for contamination even in remote areas [17,18]. When in its double-charged ionic form, Hg is highly soluble and can interact with naturally occurring organic material [19,20]. Other inorganic forms, such as chalcogenides, are more lithogenic and assumed to have lower bioavailability [21]. The primary environmental risk is attributed to methylmercury (MeHg), a respiration byproduct produced by sediment bacteria [22]. MeHg is known to readily bioconcentrate in organisms and subsequently bioaccumulate through the food web, leading to elevated concentrations in high-trophic-level predatory organisms [23].

Given the pronounced effect of the surrounding environment on Hg behaviour, numerical models are a widely accepted tool to support its monitoring and survey data, inform environmental assessments, and forecast large-scale spatiotemporal compound fluxes [24]. Fugacity-based environmental modelling was originally used to simulate the behaviour of a single compound in increasingly complex environments [25]. Fugacity describes a chemical’s ‘escaping tendency’ from a specific medium, which is influenced by site-specific parameters as well as the analytes’ physicochemical properties [26]. The construction of fugacity mass-balance models has enabled researchers to estimate regional loadings and identify pollution sources [27]. More recently, the mercury-specific ‘Hg Environmental Ratios Multi-media Ecosystem Sources’ (HERMES) model was developed [26]. This model can simulate the environmental fate of multiple Hg species and has been used in a variety of field studies to compute dynamic fluxes in large Canadian and Kazakhian lakes [26,28,29,30].

The environmental quality of the SoA has been degraded by anthropogenic activities [3,31]. The anthropogenic input reached its maximum levels in the 1980–1990s, with the reported pollution levels far exceeding the maximum permissible concentrations for fishery reservoirs in the water and sediment of the SoA and its tributaries [4,5,6,32]. More recent monitoring efforts have revealed a significant decrease in industrial inputs [4,6,10]. However, legacy pollution is expected to be recalcitrant, and chronic low-level pollution has the potential to bioaccumulate through the food web.

The aim of this study was to gain insights into the environmental Hg cycle in the SoA by constructing a mass balance model based on regional data. To achieve this, Ukrainian, Russian, and international literature were scanned for relevant model input variables. The computed values were then compared against reported environmental concentrations of Hg.

## 2. Materials and Methods

### 2.1. Theory

Fugacity describes the direction of interphase mass transfer to achieve chemical equilibrium [33]. The proportionality of the concentration of a compound (*C*) in a phase to its partial pressure or fugacity (*f*) is given by the *Z*-value (*Z*) in the unit mol/m^3^·Pa.

(1) C=Z∗f

*Z* denotes a substance- and solvent-specific solubility and is used to relate the concentration to the fugacity for different solvents. However, as the molar volume of a solvent is dependent on its molecular weight, environmental phases become difficult to simulate, thus warranting the use of empirical partitioning coefficients, *K*_12_, defined as follows:

(2) $$K_{12}=\frac{C_{1}}{C_{2}}=\frac{Z_{1}f}{Z_{2}f}=\frac{Z_{1}}{Z_{2}}$$

At equilibrium, the fugacities of two phases form a ratio of two *Z*-values. According to Mackay et al. [25], the fugacity of a substance in a simple mass balance model (a closed system in equilibrium) assuming defined phase volumes (*V*) and molarities (*M*) of a single compound with specific *Z*-values is given by

(3) $$f_{i}=\frac{M}{\sum \left(V_{i}Z_{i}\right)}$$

To produce a dynamic rather than a steady-state model, *D*-values [mol/h] can be introduced that define rate constants or rate coefficients for transport or transformation processes (*N*).

(4) N=D∗f

Total fluxes between compartments may therefore be broken down to sums of unidirectional transport processes. By combining advective and partitioning parameters, increasingly complex mass balance models can be constructed [25]. For compounds lacking a vapour pressure or general interphase partitioning, a fugacity analogue called ‘aquivalence’ may be applied [34]. For a detailed derivation and application of fugacity/aquivalence models, see Diamond et al. [35].

As previous fugacity-based models were designed to simulate the environmental fate of single organic pollutants or pollutant families with similar physicochemical properties, Toose and Mackay developed a multi-species mass balance model capable of modelling interconverting pollutants with constant species ratios [36]. To achieve this, a conventional transformation and intermedia transport rate is expressed for a single species and a multiplier for individual subspecies is deduced. for a single species, and a multiplier for individual subspecies is deduced. The newly formed combined rates of all subspecies (*D*-values) are calculated as the product of a “mother compound” and the combined multiplier (*R*).

(5) $$R_{tot}=\left(1+R_{2}+R_{3}+R_{4}\ldots +R_{i}\right)$$

At constant compartment-specific species ratios, the transformation rates of subspecies can be derived from the total *D*-value of a “mother compound”, resulting in a consistent mass balance for all species. The HERMES model combines the aquivalence principle with the multiplier method to calculate compartment-specific fugacities of the mother compound: Hg^0^. A detailed description and derivation of the HERMES model can be found in a study by Ethier et al. [26].

### 2.2. Site Description and Model Input Variables

**Figure 1:**
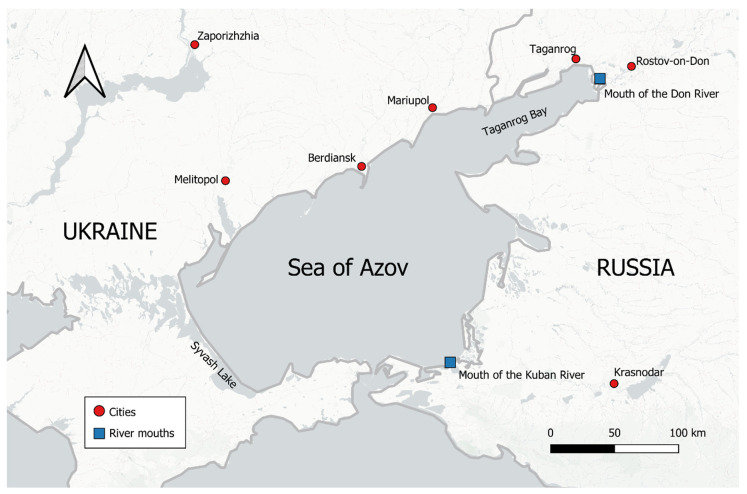
Geographical extent of the Sea of Azov. Red dots indicate large cities, and blue rectangles indicate river mouths.

The Sea of Azov (SoA) is an inland shelf sea bounded by Ukraine to the west and Russia to the east, with a connection to the Black Sea by the Strait of Kerch in the south (Figure 1). The SoA covers an area of 39,000 km^2^, with an average depth of 7 m (max. 14 m) and most bays only reaching depths of about 1 m [31]. The water balance of the SoA comprises riverine inflow, wet precipitation and evaporation, and the inflow and outflow from and into the Black Sea. About 90% of all riverine inflow is discharged from the Don and Kuban Rivers, which supply 22 and 11 km^3^/yr of water with high amounts of total suspended solids (TSS, 18.1 and 125 mg/L, respectively [37,38]). The SoA receives on average 397 mm of rain annually combined with large amounts of airborne particulate matter (approx. 9 and 15 µg/m^3^ for PM_2.5_ and PM_10_, respectively [31,39]). This constitutes a water influx of 15.5 km^3^/yr, while evaporation removes 35 km^3^/yr. The total water discharge into the Black Sea constitutes around 53–55 km^3^/yr, while the total inflow ranges between 36 and 38 km^3^/yr [31]. This mixing of riverine and marine water results in a large salinity gradient (2–12 psu) and high TSS concentrations over the whole sea (average 19.1 mg/L [31,40]), with an average organic carbon content of 16.5% [40]. Due to the large discharge of nutrients and sediment (~25.8 mg/L TSS), the river deltas not only serve as spawning grounds for a variety of commercially valuable fish species but are also pollution hotspots [31,37,41]. Despite the high sedimentation rate of 1.66 g m^−2^ day^−1^ and low resuspension rate (64%), bottom sediments contain only 2.42% organic carbon. This has been attributed to riverine TSS being dominated by inorganic constituents with comparably low organic carbon content (4.6%) [37,42,43]. The shallow depth of the SoA facilitates large seasonal variations in water temperatures, ranging from 2.9 to 25.5 °C (average 13.3 °C), without a thermocline throughout most of the watershed [44,45]. A comparative description of the SoA can be found in a book by Kosarev et al. [31].

The main influx of Hg into the SoA is via riverine input. The total Hg concentrations in the Don River averaged 520 ng/l between 1979 and 2017 [10]. The maximum concentrations in the Kuban River were recorded in 1997–98 (200 ng/L); however, more recent measurements suggest a decrease to 10 ng/L [6]. Mercury concentrations in the Black Sea average 9.5 ng/L in surface offshore waters [31]. Finally, Hg concentrations in rainwater over the SoA average 240 ng/L [46]. The abovementioned data were used to populate the HERMES model to construct a mass balance of Hg for the SoA. While the inbuilt Hg species ratios of the original model were not modified, the volatilisation mass transfer coefficient of Hg was increased to better approximate the size of the SoA and the prevailing windspeed (7.5–9 m/s) [31,47].

### 2.3. Assumptions and Limitations

Given the limited available data on Hg loadings and concentrations for the region, no further parametrisation or compartmentalisation of the model was performed for this study, as the increased complexity may have impacted its overall reliability. Additionally, a hydrodynamic steady state was assumed to balance the sediment and water budgets [48]. The SoA has a significant salinity and suspended solids gradient due to the impact of riverine discharge, which also affects Hg concentrations in the sediment and water column [49]. As the use of the HERMES model in this study is intended to approximate reported values and estimate currently unknown, undocumented, or unconsidered Hg emissions and loadings, further increases in the spatiotemporal resolution were deemed unnecessary.

To contextualise the current findings, scientific publications are cited where possible. However, due to a general scarcity of environmental information on the region, data from non-governmental organisation reports and non-peer-reviewed conference articles had to be included where no peer-reviewed scientific articles could be obtained. Those data were quality-controlled by assessing the documentation of analytical methods, evaluating the authors and publishers, and estimating the overall impact of the associated uncertainty. Data and claims derived from these sources do not conform to the standards of peer-reviewed scientific publishing, but they provided the context necessary for the present work. Their use within this study was chosen carefully and mainly served the purpose of appraising the observations and computed data in this study.

## 3. Results and Discussion

Using the reported average Hg concentration in rainwater as well as the annual precipitation rate over the SoA area, a wet deposition input of 3716.1 kg/yr was calculated. To approximate this influx, the atmospheric Hg concentration in the HERMES model was increased to 6.0 ng/m^3^. Further, Fedorov et al. stated that 28% of the total Hg (tHg) influx reached the SoA via precipitation [46]. Considering this, the total annual input into the SoA would comprise 13,271.1 kg/yr. Taking the riverine inflow of 9059.7 kg/yr, calculated from the inflow of the Don and Kuban Rivers, into account, Fedorov et al. underestimated the total influx by only 495.5 kg/yr [46]. Given the large variability in environmental parameters and the uncertainty associated with the reported data, this mass was considered negligible.

**Figure 2:**
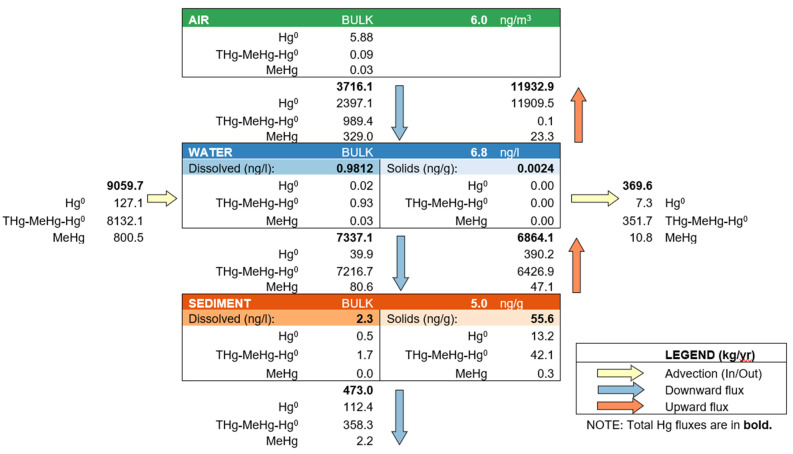
Computed total mercury (THg) concentrations using the Sea of Azov mass balance model.

Species are given as elemental mercury (Hg^0^), methylmercury (MeHg), and residual mercury (THg-MeHg-Hg^0^). Considering all the currently documented and approximated Hg loadings, the modelled compartment-specific tHg concentrations amounted to 6.8 ng/L and 55.6 ng/g dw for water and sediment, respectively (Figure 2). The primary efflux was the surface gas evasion (11,932.9 kg/yr), while the discharge in the Black Sea and sediment burial were 369.6 kg/yr and 473.0 kg/yr, respectively. While the water and sediment concentrations underestimated empirically measured offshore concentrations (Table 1), they were comparable to the most recent average concentrations reported by Korablina et al. [4] and Kuznetsov et al. [5], respectively. The computed atmospheric Hg concentration of 6.0 ng/m^3^ (Figure 2) matched average concentrations reported for remote areas on the European continent [50]. It should be noted that the compartment concentrations derived in this study represent an average value for the entire modelled system and do not account for the spatiotemporal heterogeneity caused by contamination hotspots and concentration gradients observed in empirical surveys (Table 1).

The atmospheric Hg pollution adjacent to the SoA is heavily impacted by local industry. In their assessment of pollution in the Donets basin, Panov et al. reported an atmospheric Hg concentration of 25–30 ng/m^3^ in Donetsk city and 300–1000 ng/m^3^ near a mercury refinery [2]. While these are point-source emissions, studies have documented the spatial dispersion of point-source-emitted gaseous elemental Hg (GEM) over adjacent areas, resulting in elevated atmospheric Hg concentrations comparable to this study [28]. The HERMES model used in this study focused on GEM as the main species in the atmospheric compartment; however, regional airborne particulate matter is known to carry significant Hg loadings (53 ng/g) [54].

Gas evasion is directly dependent on water temperature and the air/water mass transfer coefficient, which for GEM can range over two orders of magnitude depending on wind exposure and speed [55,56]. Recently, Zhang et al. used a coupled atmosphere–land–ocean model to update global Hg budgets [57]. Their results suggest a significantly higher surface gas evasion from ocean re-emissions than previously estimated by modelling studies. Here, re-emission from the SoA’s water was the dominant efflux in the mass balance model (90%). Due to the spatiotemporal variability in water temperature and wind speeds over the SoA, the precision of this estimation cannot be critically appraised; however, it provides evidence towards significant volatilisation in a large aquatic system.

As documented in Table 1, Hg concentrations in water and sediment are highly dependent on the sampling year and location. The values provided by Korablina et al. [32] denote averages recorded between 1986 and 1992 and 1993 and 2005, respectively, which far exceed more recent concentrations (10 ng/L) reported for 2018 in offshore waters [4]. Using the method by Ethier et al. [30], a residence time of 3.2 years for Hg loadings was computed, which underestimates the turnover reported by Korablina et al. [4]. This might be affected by the high surface gas evasion rates.

Assessments of bottom sediments in the SoA reported potentially toxic element concentrations to either not or barely exceed maximum permissible concentrations [58,59]. However, the effectiveness of sediment and water quality guidelines in safeguarding humans has been called into question [60]. The sediment concentrations produced in this study underestimate the reported values for offshore waters [5]. Given the severity of legacy pollution, the model might not be able to consider existing loadings in surface sediments.

### 3.1. Environmental Implications

The modelled sediment concentrations (Figure 2) fall below several international marine sediment quality guidelines, which estimate either no or a low risk of adverse effects in benthic organisms [60,61,62]. However, previous studies on the effects of comparable sediment concentrations reported increased Hg contents in benthic molluscs that exceeded environmental quality standards [63,64]. Furthermore, the high sedimentation intensity reported by Berdnikov and Sorokina [43] may lead to the formation of anoxic sediments, which in turn can mobilise large amounts of methane, hydrogen sulphide, gaseous elemental Hg, and other sequestered pollutants that accumulate in commercially important fish species [4,51]. Comparable values of the water column concentrations computed in this study (Figure 2) have been reported to result in bioaccumulation in high-trophic-level organisms [65]. Similar studies in the SoA reported increasing Hg concentrations in Roach (*Rutilus heckeli*) between 1992 and 2012 (max. 0.1 µg/kg) and decreasing levels until 2018 [4]. Still, fish stocks are suffering a catastrophic collapse, mainly driven by illegal fishing and environmental pollution [66,67]. Finally, the SoA provides a habitat for a distinct population of harbour porpoise (*Phocoena phocoena relicta*), which was listed as an endangered species due to its declining population size [68]. Aquatic predators are known to accumulate Hg, resulting in a range of unspecific adverse effects [23,69], which could threaten the stability of the ecosystem.

### 3.2. State of Mercury Pollution in the SoA Region

The paucity of and variability in the provided data preclude a quantitative appraisal of Ukrainian tHg emissions. Although the relevant emissions data documented under the European Monitoring and Evaluation Programme (EMEP) date back to 1991, these data are not adopted by other governing authorities. A report by the United Nations Economic Commission for Europe (ECE) estimated tHg emissions to be more than 10 times higher for the year 2003 [70]. This is further supported by other researchers who estimated emissions to be higher than the EMEP data by factors of 1.17–2.4 for the years 2013 and 2006, respectively [71,72]. The absence of dependable tHg emission data is in part due to a lack of continuous online atmospheric Hg monitoring, which has also resulted in there being no official data on Hg emissions from coal-fired power plants [70,71].

The uncertainty surrounding Hg emissions propagates into monitoring water, soil, and air quality. The dominant sources of pollution are coal-fired power plants, mercury refineries, and organomercury pesticides. Donbas basin coal is known to be uniquely contaminated with Hg deposits, with concentrations four times higher than the US average (0.68 ppm vs. 0.17 ppm [73]). During the last 200 years, approximately 10 billion tons of this coal were extracted, resulting in severe regional soil and water pollution [73,74,75]. The high degree of coal-associated regional Hg emissions and resulting regional contamination were even classified as a unique biogeochemical province by Babaev et al. [75]. This chronic exposure has resulted in significantly higher resident morbidity in comparison with the rest of the country [2]. However, soil surveys have revealed high spatiotemporal variability in Hg contamination. While some authors found hotspots in the vicinity of industrial sites and cities (19–8800 mg/kg [2,74]), others reported below-maximum permissible concentrations in some topsoils and watersheds [76].

Although the UN report on Hg releases from the Russian Federation details reported emissions, the authors state that the official statistics do not cover all sources of Hg emissions, even point sources, and that, in most cases, data provided by individual enterprises are based on calculations, not empirical measurements [77]. According to the report, Hg emissions from coal-fired utility plants are estimated to reach a maximum of 8200 kg/year, emissions to surface water bodies are expected to reach a maximum of 177 kg/year, and emissions to the atmosphere are expected to reach 2900 kg/year. Indeed, more recent assessments of the levels of heavy metals in Russian rivers have concluded a decreasing trend in mercury concentrations [6,9,10,78] along with permissible levels in soil [79]. Apart from lower emissions to water, these changes in water concentrations were attributed to sedimentation in reservoirs, which creates an additional threat of benthic methylation and subsequent biotic uptake [80]. Moreover, despite evidence of severe legacy Hg contamination in the Krasnodar and Rostov-on-Don regions as well as the Don and Kuban Rivers [6,10,12,31,46], Tarasova et al. assessed the extent of Hg pollution in the most adjacent regions to the SoA (Belgorod, Rostov, and Voronezh) and found it to not exceed the maximum acceptable concentrations [81].

Russia’s invasion of Ukraine in February 2022 has exacerbated existing environmental pollution [82,83]. In July 2022, a maximum concentration of 590 ng/L of Hg was recorded in the Sukhy Torets River, which ultimately flows into the SoA [84]. A soil survey led by the Organisation for Security and Co-operation in Europe (OSCE) covering areas directly affected by hostilities documented elevated Hg concentrations twice as high as background concentrations [85]. The most probably source is for this contamination is artillery ammunition [85], as well as the flooding and destruction of mines and mine waste disposal sites [83]. Existing studies on the impact of war-related heavy metal contamination reveal a lasting effect on the local population, including birth defects and chronic health complications [86].

## 4. Conclusions

Although the Hg concentrations in Russian rivers are falling and stricter environmental legislation has been put in place, the Sea of Azov region continues to be affected by legacy pollution. The chosen modelling approach provided an accurate estimation of regional Hg dynamics, and the computed concentrations matched those reported in the literature very closely. The ongoing war will continue to worsen the environmental situation, potentially undoing conservation efforts made over the last few years. However, the policy changes implemented by both countries may accelerate environmental recovery after the end of the hostilities.

## Figures and Tables

**Table 1:** Mercury concentration data from the literature for water and sediment in the Sea of Azov. Sediment concentrations are given on a dry weight (dw) basis.

Region	Water [ng/L]	Sediment [ng/g dw]	Reference
Taganrog Bay	100	-	[51]
	260	-	[52]
Sea of Azov (offshore)	10	<100–300	[4]
	-	25–280 (mean 67)	[5]
	170–690 160–520	-	[32]
Syvash Lake	200–600	13.8	[49]
Don River mouth	-	20,000–1,100,000	[41]
Temryuk port (Kuban River mouth)	-	149,000	[53]

## Data Availability

Dataset available on request from the authors.
